# Rheology and Molecular Mechanisms of Fracturing Fluids: A Comparison of Three Thickener Types—A Case Study

**DOI:** 10.3390/gels12020172

**Published:** 2026-02-14

**Authors:** Ke Xu, Jing Long, Xu Liang, Dingwei Weng, Pinhong Zhu, Yonghang Yi, Yingxing Chen, Cunchuan Zheng

**Affiliations:** 1 China Petroleum Exploration and Development Research Institute, Beijing 100083, China; xk0929@163.com (K.X.);; 2 College of Chemistry and Chemical Engineering, Southwest Petroleum University, Chengdu 610500, China; lj73055@163.com (J.L.);; 3 Institute of Porous Flow and Fluid Mechanics, Chinese Academy of Sciences, Langfang 065007, China; 18227060495@163.com

**Keywords:** fracturing fluid, rheological behavior, structure-rheology relationship, rheological model fitting

## Abstract

To address the lack of systematic comparison regarding rheological properties and the unclear structure–property relationships among three core fracturing fluid materials including synthetic polymers, vegetable gums, and microbial polysaccharides, this study selected acrylamide-based polymers, hydroxypropyl guar gum and xanthan gum as the representative systems. The steady-state viscosity, rheological curves, thixotropy, viscoelasticity, and temperature-shear resistance of the three samples were systematically characterized at concentrations ranging from 0.1 to 0.7 wt% using an MCR301 rotational rheometer. The outcomes indicate that the structural strength values of all three materials increase with rising concentration, but their rheological behaviors and stability differ significantly due to distinct molecular structures. The acrylamide-based copolymer forms a temporary network via weak hydrogen bonds (amide-carboxyl or amide-amide) and physical entanglements, exhibiting thixotropy and a stress pre-elastic response. The most significant effects occur at 0.7 wt%, with a thixotropic loop area of 2.874 Pa·s^−1^ and a stress overshoot of 4.97 Pa.; hydroxypropyl guar gum has insufficient thermal stability and poor heat resistance. Its viscosity retention rate is as low as 31%, and it always exhibits a solution-type rheological property of G′ < G″; the xanthan gum exhibits elastic gel properties with tanδ < 1 due to its double-helix molecular structure. It has excellent temperature shear tolerance and the viscosity retention value can reach up to 98.6 mPa·s. Two mathematical models were established and demonstrated strong applicability: a modified Carreau model for flow curve fitting yielded a coefficient of determination (R^2^) greater than 0.95, enabling accurate description of fluid-type transitions; a four-parameter equation for temperature–shear resistance curves also achieved an R^2^ above 0.95, effectively characterizing viscosity evolution with temperature.

## 1. Introduction

Hydraulic fracturing is a key technology for the efficient development of unconventional oil and gas resources [1,2,3]. Its core lies in creating an artificial fracture network through high-pressure fluid injection to improve reservoir permeability and single-well productivity. As the pressure transmission medium that also affects the transportation and placement of proppants, fracturing fluid performance determines the success or failure of fracturing operations [4,5,6,7,8]. Water-based fracturing fluids have become the mainstream application due to their wide availability, low cost and environmental friendliness [9]. As the core component of water-based fracturing fluids, thickeners are high molecular polymers with specific structures [10,11,12], which regulate the rheological behavior of the system through molecular entanglement or network structure formation. The molecular structure of thickeners directly determines the key performance of fracturing fluids such as steady-state viscosity, thixotropy, temperature and shear resistance, and further affects fracture propagation, proppant-carrying efficiency and construction safety [13,14,15]. Therefore, clarifying the structure–property relationship between the molecular structure and rheological properties of thickeners and establishing a systematic formulation screening and evaluation mechanism are crucial for improving the scientificity and engineering adaptability of fracturing fluid design.

At present, the water-based fracturing fluid thickeners widely used in engineering can be mainly divided into three categories: synthetic polymers, plant gums, and exopolysaccharides [16,17,18]. Represented by polyacrylamide and its derivatives, synthetic polymers feature a highly designable molecular structure, good solubility, a wide range of performance regulation, and relatively controllable costs. They are suitable for medium-deep, medium-high temperature and low-permeability oil and gas reservoirs, and are widely applied in slickwater and low-viscosity fracturing systems [19,20]. Plant gum thickeners are typified by guar gum and its modified products, and also include natural plant extracts such as konjac gum, sesbania gum, and locust bean gum. With active groups-rich molecular chains and excellent water solubility, they can be cross-linked into weak gels, presenting the advantages of environmental friendliness, cost-effectiveness, and good viscosity enhancement and proppant suspension performance. Adapted for medium-high viscosity cross-linked fracturing fluids, they have been maturely applied in the fracturing of conventional oil and gas reservoirs [21,22,23]. Exopolysaccharide thickeners are represented by xanthan gum, including gellan gum, curdlan, etc., which are produced by microbial fermentation. Endowed with a unique structure, excellent water solubility, rigid molecular chains and outstanding gel stability, they are suitable for fracturing fluid systems in deep formations with high temperature and high pressure as well as under complex working conditions [24,25,26,27].

Current research on the rheological properties of fracturing fluid thickeners mainly focuses on the steady-state viscosity and flow behavior of single systems, thixotropy and structural recovery under shear, and dynamic viscoelasticity and proppant-carrying performance [28,29,30]. However, most studies concentrate on a single material or index, lacking systematic cross-category comparisons under a unified test system. The understanding of the intrinsic correlation between molecular structure and macroscopic rheology remains inadequate, which hinders the quantitative screening and optimization of thickeners for engineering operating conditions.

In summary, this study takes three types of core thickening materials for fracturing fluids—acrylamide-based polymers, plant gums, and exopolysaccharides—as the research objects. Combining rheological and microstructural analyses, it systematically tests their rheological properties under multiple conditions and quantifies the performance differences, reveals the regulatory laws of molecular structure on rheology, clarifies the correlation between concentration and molecular chain network structure, establishes a new rheological model, and explores the intrinsic causes of the differences in rheological behaviors. This work provides theoretical guidance and data support for the optimal selection of thickeners under engineering operating conditions.

## 2. Results and Discussion

### 2.1. Steady-State Viscosity

**Figure 1:**
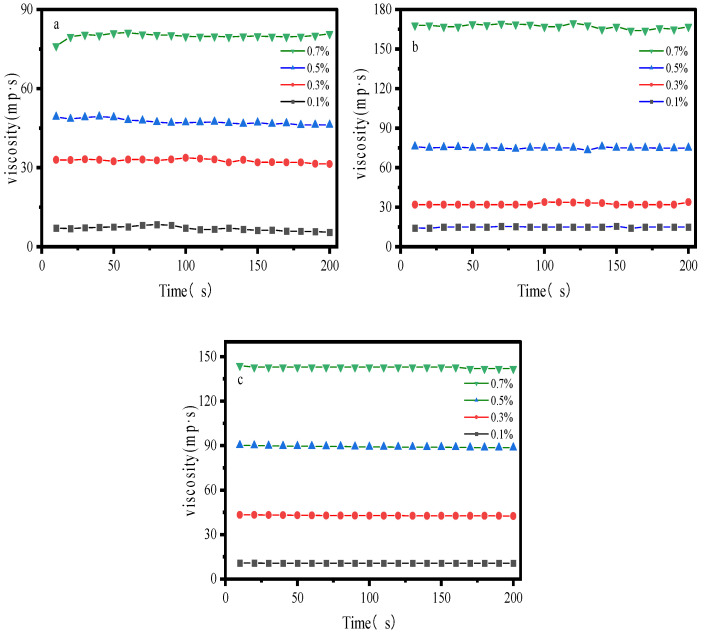
Steady-state viscosity measurement curve: (**a**) AM/AA/AMPS; (**b**) HPG; (**c**) XG.

As illustrated in Figure 1, under a constant shear rate of 100 s^−1^, the steady-state viscosities of all three samples increased monotonically with increasing concentration. The effect of shear duration on viscosity was negligible, indicating good stability of the three systems. At 0.1 wt%, both hydroxypropyl guar gum and xanthan gum exhibited similar viscosities of approximately 10 mPa·s. At 0.3 wt%, xanthan gum displayed the highest viscosity, reaching 42 mPa·s. At 0.7 wt%, both hydroxypropyl guar gum and xanthan gum demonstrated significantly elevated steady-state viscosities exceeding 140 mPa·s. Hydroxypropyl guar gum exhibited a dramatic more than twofold viscosity increase between 0.5 wt% and 0.7 wt%. Hydroxypropyl guar (HPG) molecules are rich in hydroxyl groups and can form a high-density network of intermolecular hydrogen bonds and physical entanglements in aqueous solution. The system maintains strong structural resistance under steady shear, resulting in the highest steady shear viscosity. Acrylamide-based polymers have no branched structure and their molecular chains lack ordered entanglements. Therefore, they exhibit the lowest viscosity at all concentrations, with an average value of approximately 80 mPa·s at the highest concentration and about 7 mPa·s at the lowest concentration.

### 2.2. Flow Curves

**Figure 2:**
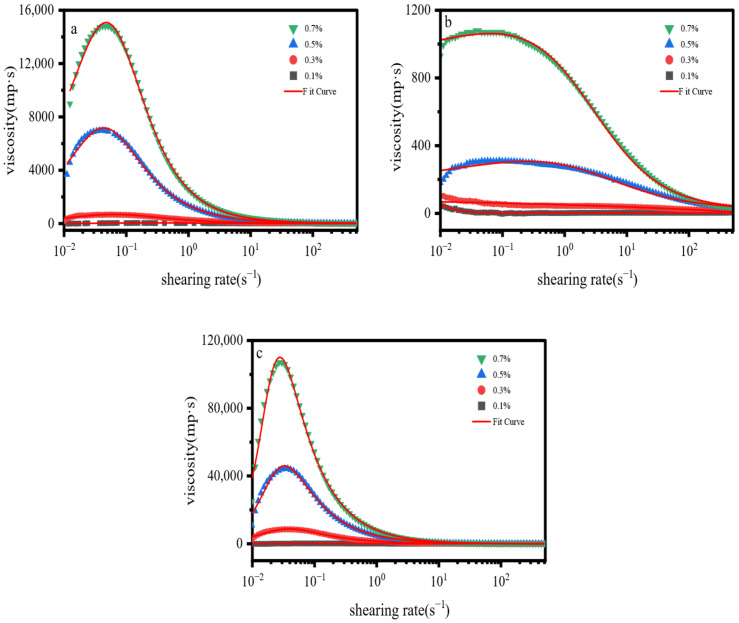
Flow Curve: (**a**) AM/AA/AMPS; (**b**) HPG; (**c**) XG.

As illustrated in Figure 2, at low shear rates (0.01–0.1 s^−1^), the viscosities of all three samples increased to varying degrees, indicative of weak dilatant (shear thickening) behavior [31,32]. This trend was most pronounced for the acrylamide based polymer and xanthan gum. Following the attainment of peak viscosity, all samples exhibited a significant decrease in viscosity with increasing shear rate, consistent with pseudoplastic (shear thinning) behavior [33]. We hypothesize that at low shear rates, intermolecular weak hydrogen bonding interactions form transient associative networks, which causes an increase in viscosity. With the increase in shear rate, these associative networks are gradually disrupted, leading to the alignment of polymer backbones along the flow direction and thus a significant decrease in viscosity.

In previous studies, researchers have mostly adopted model equations such as the Carreau model and the power-law model [32,33] to analyze flow curves and describe the rheological behavior of fluids, which mainly characterize a single behavior of either shear thickening or shear thinning. However, the special rheological property of first shear thickening followed by shear thinning observed in this study cannot be accurately depicted by these classical rheological equations, as they fail to fit both the thickening and thinning regions simultaneously. Therefore, we established a new model equation (Equation (1)) based on the Carreau model, introducing additional characteristic parameters including the critical shear rate ${\dot{\gamma }}_{c}$, thickening index *b* and thinning index *d* for fitting such characteristic curves. The results (Table 1) show that this proposed equation achieves good fitting effects for all three materials, which verifies its strong applicability.

(1) $$\eta =\eta_{\infty }+\left(\eta_{0}-\eta_{\infty }\right)\times \frac{e^{A}*{\dot{\gamma }}^{b}}{1+{\left(\frac{\dot{\gamma }}{{\dot{\gamma }}_{c}}\right)}^{d}}$$

where *η* is the apparent viscosity (mPa·s); $\dot{\gamma }$ is the shear rate (1/s); *η*_0_ is the zero-shear viscosity, corresponding to the viscosity as the shear rate approaches zero; *η_∞_* is the infinite-shear viscosity, representing the asymptotic viscosity at high shear rates; *A* is the viscosity change intensity; *b* is the shear-thickening index; ${\dot{\gamma }}_{c}$ is the characteristic shear rate, which corresponds to the shear rate at the viscosity peak; and *d* is the shear-thinning index. Here, *d* > *b* > 0.

### 2.3. Thixotropy

**Figure 3:**
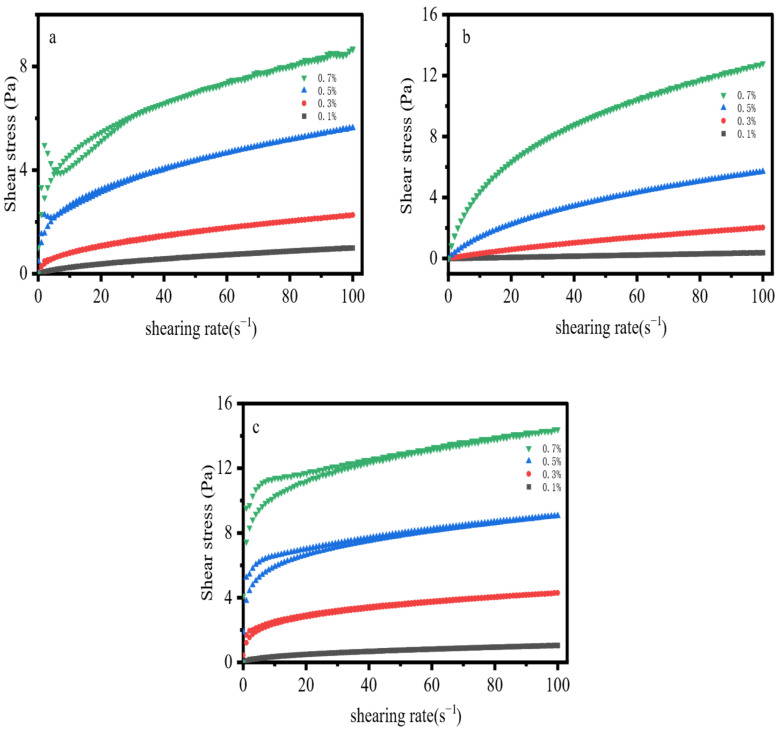
Thixotropy: (**a**) AM/AA/AMPS; (**b**) HPG; (**c**) XG.

As can be seen from the thixotropy measurement results (Figure 3), the acrylamide-based polymer exhibited thixotropic behavior at concentrations of 0.5% and 0.7%, with the corresponding hysteresis loop areas listed in Table 2. This concentration range was accompanied by a typical stress overshoot phenomenon [34,35,36], it originates from the combined effects of the flexible main chain, hydrogen bonding, and strong anionic electrostatic repulsion. with peak overshoot values of 2.3 Pa and 4.97 Pa, respectively. When the concentration of the acrylamide-based polymer increased from 0.5 wt% to 0.7 wt%, both the hysteresis loop area and the stress overshoot value increased simultaneously, which was attributed to the corresponding rise in the density of molecular chain entanglement points and the enhanced weak hydrogen bonding interactions with increasing concentration. No thixotropic behavior was observed at low concentrations (<0.5 wt%) due to insufficient entanglement caused by the ultra-low concentration. Hydroxypropyl guar gum (HPG) showed no significant thixotropy at all tested concentrations, with no obvious hysteresis loops detected. Xanthan gum exhibited the strongest thixotropy; at a concentration of 0.7 wt%, the hysteresis loop area of xanthan gum reached 30.36 Pa, which was more than ten times that of the acrylamide-based polymer at the same concentration, demonstrating its far superior network structure strength. The analysis of thixotropy indicated that the network structure strength of the three materials followed the order: XG > AM/AA/AMPS > HPG.

### 2.4. Viscoelasticity

(1) Strain Sweep

**Figure 4:**
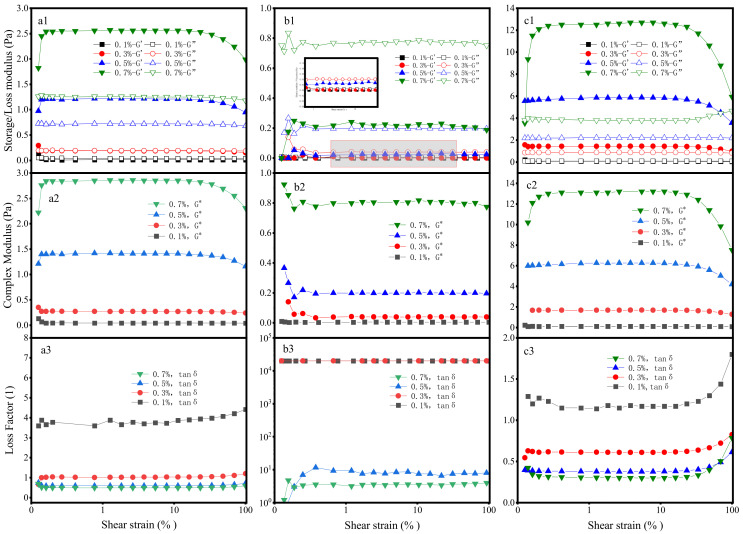
Strain Sweep: (**a**) AM/AA/AMPS; (**b**) HPG; (**c**) XG. (**a1**–**c1**) Storage/Loss modulus; (**a2**–**c2**) Complex Modulus; (**a3**–**c3**) Loss Factor.

The observed viscoelastic disparities are intrinsically linked to the molecular architectures and entanglement states of the polymers [37,38]. As depicted in Figure 4, the acrylamide-based polymer exhibits G′ > G″ across all concentrations, indicative of a predominantly elastic response. Xanthan gum also displays gel-like characteristics. Owing to its rigid helical structure and dense side-chain branching, xanthan gum forms a robust entanglement network, resulting in a viscoelasticity that is 4–5 times greater than that of the acrylamide-based polymer at equivalent concentrations. Conversely, guar gum, characterized by weak entanglements between its linear chains, exhibits both G′ and G″ moduli of less than 1 Pa with G′ < G″, confirming a typical solution-like behavior. A comparison of the complex modulus (G*) reveals the overall viscoelasticity of the three materials follows the order of XG > AM/AA/AMPS > HPG, which is consistent with the thixotropy test results.

Furthermore, the viscoelasticity of all three polymers increases with concentration, accompanied by a broad linear viscoelastic region (LVR). This behavior indicates that higher concentrations enhance the molecular chain entanglement density and strengthen the network structure, thereby improving the resistance to deformation.

The loss factor (tanδ) is defined as the ratio of the loss modulus (G″) to the storage modulus (G′), i.e., tanδ = G″/G′. Its value directly reflects the proportion of energy dissipation to energy storage within the material. It further reflects the differences in the fluid properties of the materials. Xanthan gum exhibits distinct gel characteristics; the tanδ value of the acrylamide-based polymer is higher than that of xanthan gum but still below 1; hydroxypropyl guar gum has the largest tanδ value among the three materials and the value is greater than 1, indicating its prominent solution-like behavior. Furthermore, the results show that in the three systems, the viscoelasticity and thixotropy of the solutions exhibit consistent variation trends; the stronger the elasticity of the system, the higher the overall viscoelasticity level and the more significant the thixotropy.

(2) Frequency Sweep

Frequency sweep experiments are governed by the principle of timescale correspondence—high frequencies probe short time scales (local segmental dynamics), while low frequencies access long time scales (global chain relaxation or network reorganization). The disparate viscoelastic responses of the three samples originate fundamentally from differences in their molecular architectures and aggregate structures.

**Figure 5:**
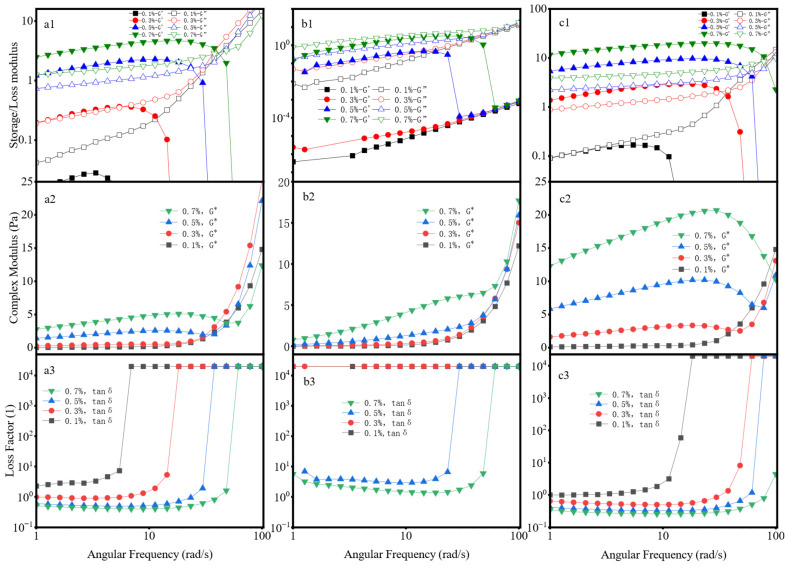
Frequency Sweep: (**a**) AM/AA/AMPS; (**b**) HPG; (**c**) XG. (**a1**–**c1**) Storage/Loss modulus; (**a2**–**c2**) Complex Modulus; (**a3**–**c3**) Loss Factor.

As illustrated in Figure 5, for the acrylamide-based polymer within the frequency sweep range, the storage modulus (G′) was less than the loss modulus (G′′) at a concentration of 0.1 wt%, where the system was dominated by viscosity and exhibited solution-like behavior. With the increase in frequency, intersections between G′ and G′′ occurred at angular frequencies of 5.46, 23.4 and 37.9 rad/s for the concentrations of 0.3 wt%, 0.5 wt% and 0.7 wt%, respectively. These intersections are the characteristic frequency points of the solution [39,40], corresponding to the reciprocals of the relaxation time of the solution system (Table 3). In the concentration range of 0.3 wt% to 0.7 wt%, the relaxation time of the solution decreased from 0.183 s to 0.026 s, all falling within the moderate relaxation time range. This indicates that the intermolecular response and structural reconstruction of the system are balanced, enabling the controllable switch between elastic behavior at rest and viscous behavior under shear. Xanthan gum exhibited a shorter relaxation time, which demonstrates its excellent resistance to dynamic shear and suitability for high-frequency dynamic working conditions. For HPG, G′ was consistently lower than G′′ at all tested concentrations regardless of frequency variations, with the system dominated by viscous flow.

The loss factor (tanδ) follows the order hydroxypropyl guar gum (HPG) > acrylamide-based polymers (AM/AA/AMPS) > xanthan gum (XG). HPG lacks an elastic network and keeps tanδ > 1, showing the strongest energy dissipation. Acrylamide-based polymers form concentration-dependent weak networks, with tanδ fluctuating around unity, enabling dynamic sol–gel transition. XG constructs a rigid network via double-helix structures, maintaining tanδ < 1 in the low-frequency region and presenting the most efficient energy storage. These three materials show a gradient in rheological type, with the above tanδ order corresponding to solution, weak gel, and gel behaviors, respectively.

### 2.5. Viscosity–Temperature Dependence

**Figure 6:**
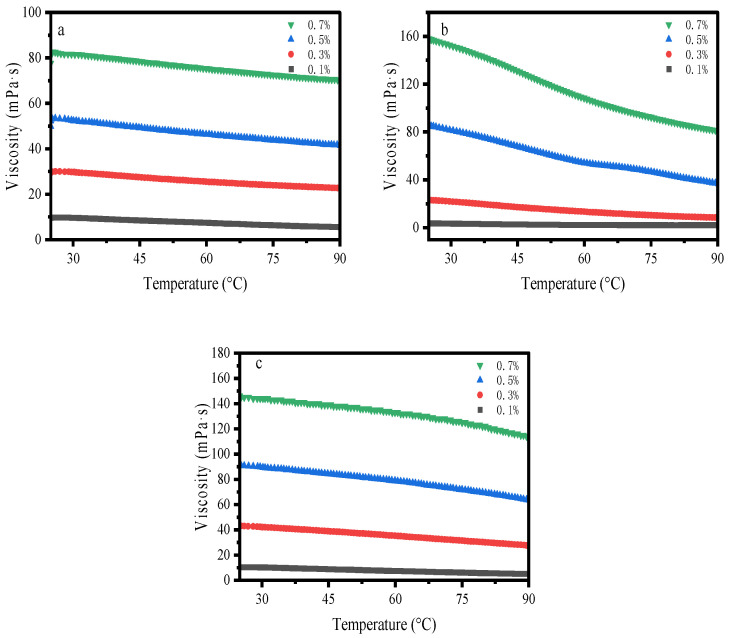
Viscosity–Temperature Curve: (**a**) AM/AA/AMPS; (**b**) HPG; (**c**) XG.

As depicted in Figure 6, under continuous shear during heating, the viscosity of all three samples decreases. This phenomenon arises from the disruption of internal structures and the intensification of molecular thermal motion at elevated temperatures, which weakens intermolecular forces such as van der Waals forces and hydrogen bonds. Consequently, the entangled molecular chains readily disentangle, leading to a reduction in internal frictional resistance and a subsequent decline in viscosity [41]. The viscosity retention rates are summarized in Table 4. The viscosity–temperature curve of the acrylamide-based polymer lies consistently at the bottom, with viscosities lower than those of the other two samples across all temperatures and concentrations. However, it exhibits the highest viscosity retention rate (0.7 wt%, retention rate 85.25%), this is attributed to the fact that polyacrylamide-based polymers exhibit low intrinsic viscosity; however, their sulfonic acid groups retard the degradation of molecular networks under high-temperature conditions through steric hindrance and hydrophilic interactions, thereby demonstrating a relatively higher viscosity retention rate. Hydroxypropyl guar gum displays a distinct thermal disadvantage, suffering significant viscosity loss. This may be attributed to the disruption of the ordered arrangement of water molecules around the polymer chains at higher temperatures; the disappearance of the hydration shell alters the molecular conformation [42], resulting in viscosity reduction. Xanthan gum maintains a high residual viscosity at all concentrations; at 0.7 wt% and 90 °C, its viscosity remains close to 120 mPa·s, which is approximately twice that of the acrylamide-based polymer at the same conditions. In terms of temperature resistance and shear resistance, the performance follows the order: AM/AA/AMPS > XG > HPG.

### 2.6. Temperature and Shear Resistance

**Figure 7:**
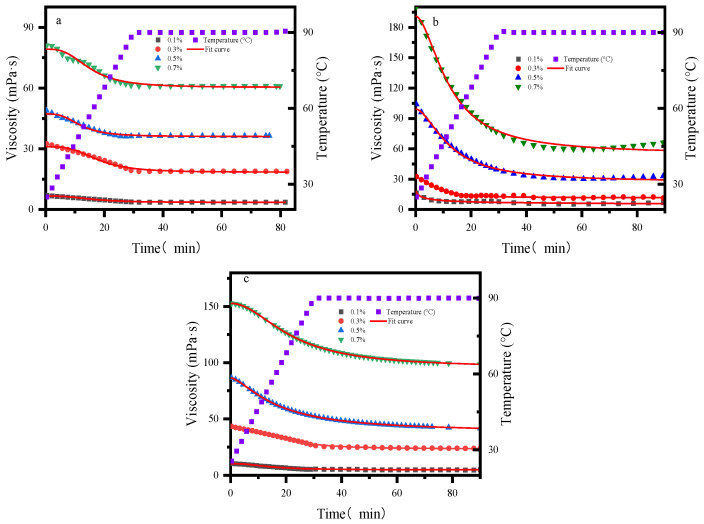
Temperature and Shear Resistance: (**a**) AM/AA/AMPS; (**b**) HPG; (**c**) XG.

As illustrated in Figure 7, all three samples exhibit a characteristic viscosity decrease followed by a plateau under continuous heating. After 40 min, the viscosity stabilizes, indicating that the system has reached a steady state under the combined effects of temperature and shear. The residual viscosity values of all concentrations after 60 min of continuous shearing at 90 °C and 100 s^−1^ are summarized in Table 5, the results show that the viscosity retention rate follows the order: AM/AA/AMPS > XG > HPG, which is consistent with the results of the viscosity–temperature curve. This further confirms the differences in temperature resistance among the three materials and provides a reference for the temperature-resistant design of fracturing fluids in terms of temperature-resistant functional groups and double-helical molecular chains. A four-parameter equation [43], Equation (2) was employed to fit the viscosity evolution during the temperature and shear resistance test, with the fitting parameters presented in Table 6. The results demonstrate that the model fits the viscosity profiles of all three systems well, with correlation coefficients (R^2^) greater than 0.95.

(2) $$\frac{\eta -\eta_{min}}{\eta_{0}-\eta_{min}}=\frac{1}{1+{\left(kt\right)}^{c}}$$

where *η*_0_ is the initial viscosity, *η_min_* is the minimum viscosity, *K* is the rate constant of structural change during the temperature and shear resistance process, and *C* is the correlation coefficient of viscosity with respect to structural changes.

### 2.7. Modulus–Temperature Profiles

**Figure 8:**
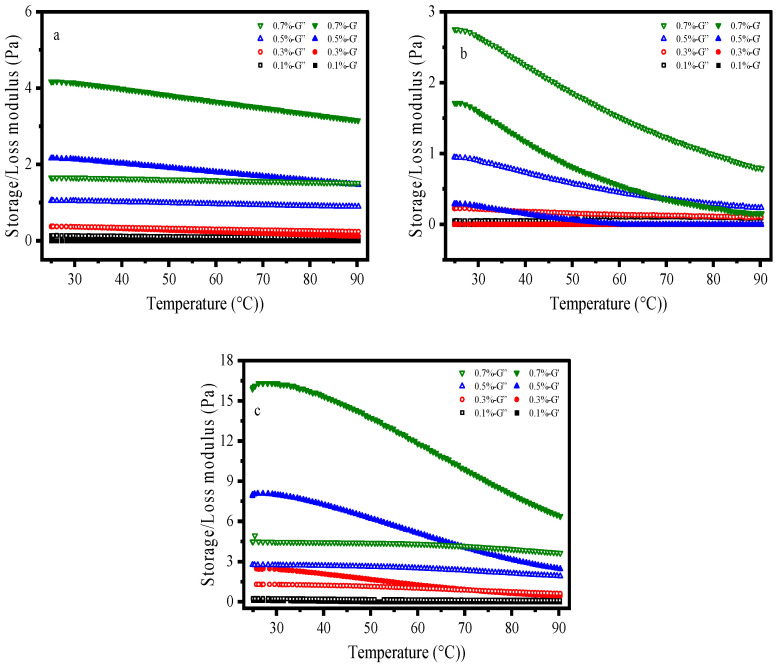
Modulus–Temperature Curve: (**a**) AM/AA/AMPS; (**b**) HPG; (**c**) XG.

As depicted in Figure 8, the acrylamide-based polymer exhibits the smallest variation in modulus, with the curves remaining relatively flat across the temperature range. At 0.7 wt%, G′ decreases marginally from approximately 4 Pa at 25 °C to 3 Pa at 90 °C, representing a mere 25% reduction. Furthermore, at equivalent concentrations and temperatures, G′ consistently exceeds G″, confirming the dominance of elastic behavior throughout the test. For instance, at 0.5 wt% and 50 °C, G ≈ 2 Pa, G ≈ 1 Pa, and tanδ ≈ 0.5, which is less than 1.

Hydroxypropyl guar gum exhibited the largest magnitude of modulus variation. At 0.7 wt%, G′ decreases substantially from approximately 1.8 Pa at 25 °C to 0.2 Pa at 90 °C, representing a dramatic reduction of 88%. Under identical concentration and temperature conditions, the system consistently remains in a viscous-dominated state characterized by G″ > G′ and tanδ > 1. Elevated temperatures significantly enhance molecular chain mobility, promoting the rapid dissociation of temporary associative structures and consequently leading to a substantial decline in modulus. Nevertheless, G″ remains greater than G′, confirming that the viscous-dominated nature of the system is preserved. At higher concentrations, the molecular chains form a denser entanglement network; upon heating, the disentanglement process is more extensive, which accounts for the larger reduction in modulus observed.

In xanthan gum solutions, at 0.7 wt%, G′ decreases from approximately 16 Pa at 25 °C to 6 Pa at 90 °C, representing a reduction of about 62%. At 0.1 wt%, G′ value drops from around 0.2 Pa at 25 °C (G′ ≈ 0.2 Pa) to 0.09 Pa at 90 °C (G′ ≈ 0.09 Pa), with a decrease of approximately 55%. Under the same concentration and temperature conditions, xanthan gum solutions exhibit G′ values significantly higher than G″, with tanδ < 1 and the lowest values among the three samples, indicative of elastic dominance. Although increasing temperature disrupts some hydrogen bonds, causing network loosening and a decrease in modulus, the double-helical structure itself possesses inherent rigidity, and the remaining entanglements are sufficient to maintain a dense network. Consequently, G′ remains substantially higher than G″, and strong elastic dominance is preserved.

### 2.8. Microstructural Analysis

**Figure 9:**
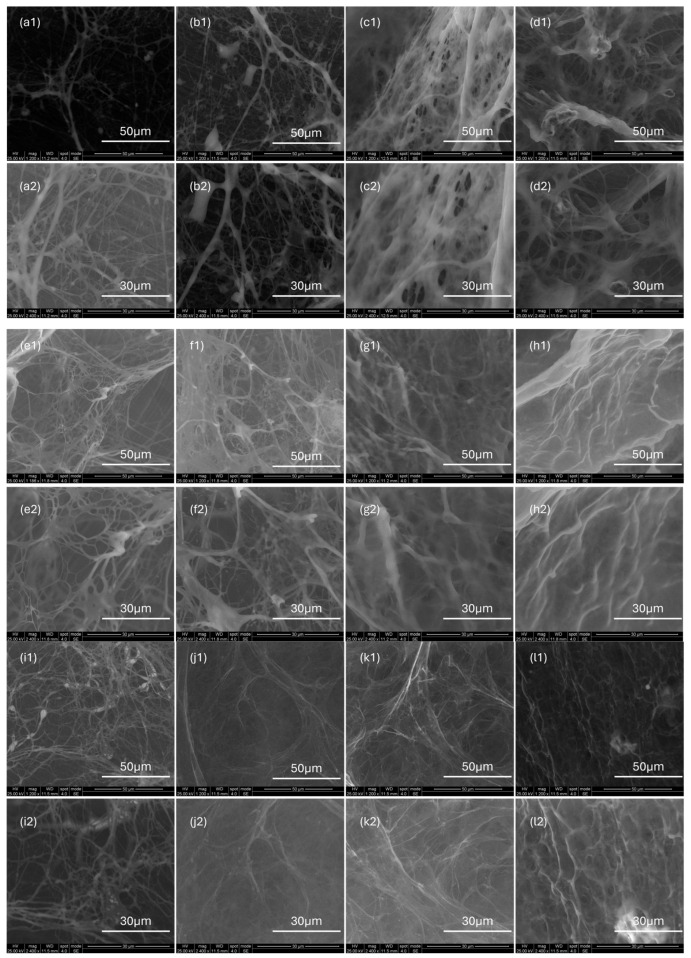
SEM images of AM/AA/AMPS, HPG and XG solutions with mass concentrations ranging from 0.1 to 0.7 wt% at different magnifications. (**a1**–**d1**) AM/AA/AMPS solutions at 1200× magnification; (**a2**–**d2**) AM/AA/AMPS solutions at 2400× magnification; (**e1**–**h1**) HPG solutions at 1200× magnification; (**e2**–**h2**) HPG solutions at 2400× magnification; (**i1**–**l1**) XG solutions at 1200× magnification; (**i2**–**l2**) XG solutions at 2400× magnification.

Scanning electron microscopy (SEM) images (Figure 9) reveal that for all three samples, an increasing concentration leads to the formation of a denser and more interconnected network morphology, characterized by a significant reduction in pore size and an increase in the number of structural skeletons. This evolution indicates that the degree of spatial overlap and entanglement between molecular chains increases with concentration. The corresponding rheological measurements demonstrate a concurrent increase in viscosity and viscoelasticity, reflecting the trend toward network densification and confirming the direct correlation between microscopic structure and macroscopic rheological behavior. It should be noted that SEM observations only reflect the morphological characteristics of the polymer solutions under fixed processing conditions; their primary role is to provide qualitative structural corroboration for the rheological results, rather than serving as a direct representation of the true molecular conformations in the original solution state.

## 3. Conclusions

This study systematically characterizes the rheological properties of three representative water-based fracturing fluid materials—polyacrylamide-based polymer (a synthetic polymer), hydroxypropyl guar gum (a plant gum), and xanthan gum (a microbial polysaccharide)—through experimental investigation and theoretical analysis. By integrating molecular structural insights, the intrinsic mechanisms underlying their rheological discrepancies are elucidated, and a suitable mathematical model is developed to describe their rheological behavior. The primary conclusions are summarized as follows:

1. Concentration acts as the key regulatory factor for the rheological properties of the three systems. With the increase in concentration, the network structure of the systems becomes increasingly dense, and all rheological parameters increase significantly accordingly. In addition, the results of strain and frequency sweep experiments show that the rheological types of the three materials present a gradient distribution, with the loss tangent (tanδ) following the order of XG > AM/AA/AMPS > HPG, which corresponds to the intrinsic rheological natures of gel, weak gel and solution, respectively.

2. The flow curves of the three materials all exhibit the rheological behavior of first thickening and then thinning. A new model equation was thus established to characterize this behavior, achieving a good fitting effect (R^2^ > 0.95). Characteristic parameters such as the critical shear rate (${\dot{\gamma }}_{c}$), thickening index (*b*) and thinning index (*d*) can be obtained through fitting. Breaking through the limitation that traditional models (e.g., Carreau, Cross and power-law models) can only fit a single rheological behavior, this model realizes the quantitative characterization of composite rheological behavior. Meanwhile, a four-parameter equation was constructed to describe the viscosity variation during the temperature and shear resistance process.

3. Core Mechanisms Underlying the Differences in Rheological Properties:

(1) AM/AA/AMPS forms weak cross-linking sites via intermolecular hydrogen bonding and electrostatic interactions, exhibiting typical viscoelastic responses such as thixotropy and stress overshoot. Its flexible carbon chains and the weak hydration capacity of hydrophilic groups result in a lower viscosity compared with the other two materials.

(2) Hydroxypropyl guar gum (HPG) features linearly arranged backbones and randomly distributed side chains, which associate only through weak van der Waals forces without forming a stable elastic network, leading to poor viscoelasticity. Additionally, its glycosidic linkages tend to dissociate at high temperatures, resulting in inferior temperature resistance.

(3) Xanthan gum (XG) possesses the strongest three-dimensional network structure among the three materials due to its unique double-helix structure, thus exhibiting excellent shear resistance, high temperature resistance, prominent viscoelasticity and strong thixotropy.

Through systematic experiments and mechanism analysis, this study overcomes the limitations of previous research and achieves breakthroughs in fracturing fluid rheology: from single-index evaluation to multi-parameter integration, from qualitative structure–activity relationship to quantitative regulation, and from classical models to customized fitting. The research results enrich the relevant theoretical system and provide precise support for material selection, formulation optimization and the molecular design of fracturing fluids.

## 4. Materials and Methods

### 4.1. Materials

The acrylamide-based polymer was prepared in the laboratory (Langfang City, China). Hydroxypropyl guar gum and xanthan gum were kindly provided by the PetroChina Research Institute of Petroleum Exploration and Development (Langfang City, China).

1. Acrylamide-based Terpolymer (AM/AA/AMPS terpolymer)

**Figure 10:**
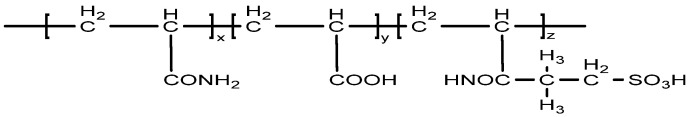
The molecular structure of AM/AA/AMPS terpolymer.

This linear macromolecule is synthesized via the random copolymerization of acrylic acid (AA), acrylamide (AM), and 2-acrylamido-2-methylpropane sulfonic acid (AMPS), resulting in a polymer architecture (Figure 10) that simultaneously incorporates carboxyl, amide, and sulfonic acid functional groups along the pendant positions of the polymer chain. The backbone is formed through the free-radical addition polymerization of vinyl moieties [44].

2. Hydroxypropyl Guar Gum (HPG)

**Figure 11:**
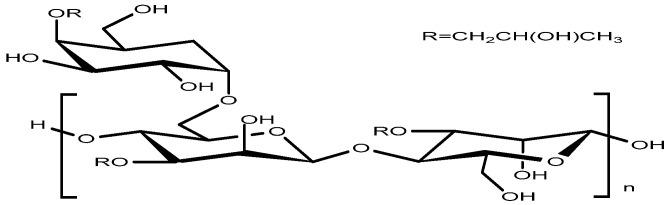
The molecular structure of hydroxypropyl guar gum.

Hydroxypropyl guar gum (HPG) is a chemically modified galactomannan derivative produced by the alkaline-catalyzed etherification of native guar gum with propylene oxide, wherein multiple hydroxyl groups along the polysaccharide backbone are substituted by hydroxypropyl moieties [45]. As a typical galactomannan, HPG features a linear backbone composed of β-D-mannopyranose residues linked via β-1, 4-glycosidic bonds, with α-D-galactopyranose units attached as side chains in a random distribution(Figure 11).

3. Xanthan Gum (XG)

**Figure 12:**
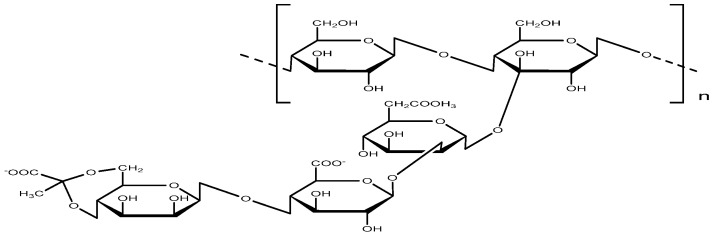
The molecular structure of xanthan gum.

Xanthan gum is an extracellular polysaccharide biosynthesized by Xanthomonas campestris, featuring a complex linear structure (Figure 12) composed of D-glucose, D-mannose, and D-glucuronic acid in a 2:2:1 molar ratio [46]. In aqueous solution, xanthan gum molecules form a highly entangled three-dimensional network, with side-chain interactions—mediated by hydrogen bonding and van der Waals forces—promoting the formation of helical and double-helical conformations [47,48].

### 4.2. Methodology

(1) Sample Preparation

A 400 mL aliquot of distilled water was measured using a graduated cylinder and transferred into the mixing chamber of a clean, dry Waring Blender. The plant gum thickener and solid additives, pre-weighed according to the specific formulation, were incorporated gradually into the vortex to mitigate the formation of fisheyes. The mixture was homogenized at 1500 r/min for 2 min, followed by agitation at 1000 r/min for an additional 3 min. Subsequent to static aging at ambient temperature for 4 h, rheological measurements were conducted.

(2) Rheological Test Procedures

Rheological characterization was performed using a Physica MCR 301 modular compact rheometer (Anton Paar GmbH, Graz, Austria). The instrument operates in both stress-controlled (CS) and strain-controlled (CR) modes, facilitating a comprehensive suite of rotational and oscillatory tests. Key technical specifications include a minimum torque resolution of 0.01 μNm, a minimum frequency of 1.0 × 10^−7^ Hz, and an angular velocity range (CR mode) of 1.0 × 10^−8^ to 1000 rad/s, with temperature control achievable from −150 °C to +250 °C. All experiments were executed via the RheoCompass software (Professional build 1.24.549) platform, leveraging the Tool master function for automatic sensor and geometry recognition to ensure optimal data integrity and reproducibility.

The rheological behavior of three polymer systems—acrylamide-based polymer, hydroxypropyl guar gum, and xanthan gum—was systematically characterized across a concentration range of 0.1 to 0.7 wt% (0.1, 0.3, 0.5, and 0.7 wt%). All measurements were conducted under isothermal conditions at 25 °C. The experimental investigation encompassed the following aspects:

① Steady-State Viscosity: A constant shear rate of 100 s^−1^ was applied to the sample for a duration of 200 s to ensure the attainment of steady-state conditions. ② Flow Curve: A logarithmic shear rate sweep was performed from 0.01 s^−1^ to 1000 s^−1^ to characterize the shear-thinning behavior of the fluids. ③ Thixotropic Loop: The shear rate was linearly ramped up from 0 s^−1^ to 100 s^−1^ over 200 s, followed by a linear ramp down to 0.1 s^−1^ over an additional 200 s. ④ Viscoelastic Properties: Strain Sweep: To determine the linear viscoelastic region (LVR), the shear strain was increased logarithmically from 0.1% to 100% at a fixed angular frequency (ω) of 1 rad·s^−1^. Frequency Sweep: The angular frequency was varied logarithmically from 1 rad·s^−1^ to 100 rad·s^−1^ at a constant strain of 1% (within the LVR) to evaluate the structural integrity of the gels. ⑤ Viscosity–Temperature Dependence: At a constant shear rate of $\dot{\gamma }$ = 100 s^−1^, the temperature was linearly increased from 25 °C to 90 °C at a heating rate of 0.54 °C/min (over 1200 s) to monitor the thermal stability of the viscosity. ⑥ Thermal and Shear Resistance Test: The sample was subjected to a constant shear rate of 100 s^−1^. The temperature was ramped from 25 °C to 90 °C over 30 min and subsequently held at 90 °C for 60 min under continuous shear. ⑦ Modulus-Temperature Profile: Under oscillatory conditions (1% strain, 1 Hz frequency), the temperature was increased at a rate of 3 °C/min from 25 °C to 90 °C to obtain the temperature dependence of the viscoelastic moduli.

### 4.3. The Establishment of the New Model Equation

To account for the observed viscosity increase at low shear rates and subsequent decrease at high shear rates, a new model equation was formulated to capture the flow behavior of the three samples. The fitting results are summarized in Table 1, and the model exhibits excellent agreement with the experimental data, with most coefficients of determination (R^2^) greater than 0.95.

I. The governing equation of the Carreau model [49] is given by

(3) $$\eta =\eta_{\infty }+\left(\eta_{0}-\eta_{\infty }\right)*{\left[1+{\left(\lambda \dot{\gamma }\right)}^{2}\right]}^{\frac{n-1}{2}}$$

The model captures the limiting viscosity behavior, with *η* approaching the zero-shear viscosity *η*_0_ (i.e., $\dot{\gamma }$ → 0) at low shear rates and the infinite-shear viscosity *η_∞_* (i.e., $\dot{\gamma }$ → ∞) at high shear rates.

*η* denotes the apparent viscosity at a given shear rate *γ*; *η*_0_ represents the zero-shear viscosity (the asymptotic viscosity at low shear rates); *η_∞_* is the infinite-shear viscosity (the asymptotic viscosity at high shear rates); *λ* is the time constant, characterizing the transition rate from low-shear to high-shear behavior; and *n* is the power-law index, which dictates the shear-thinning or shear-thickening nature of the fluid (*n* < 1 for shear-thinning and *n* > 1 for shear-thickening).

II. Model Development

Building upon the Carreau framework, a generalized model was formulated by introducing parameters that describe the viscosity change intensity, characteristic shear rate, and corresponding power-law index. The resulting equation is given by

(4) $$\eta =\eta_{\infty }+\left(\eta_{0}-\eta_{\infty }\right)*\frac{e^{A}*{\dot{\gamma }}^{b}}{1+{\left(\frac{\dot{\gamma }}{{\dot{\gamma }}_{c}}\right)}^{d}}$$

where *η* is the apparent viscosity (mPa·s); $\dot{\gamma }$ is the shear rate (1/s); *η*_0_ is the zero-shear viscosity, corresponding to the viscosity as the shear rate approaches zero; *η*_∞_ is the infinite-shear viscosity, representing the asymptotic viscosity at high shear rates; *A* is the viscosity change intensity; *b* is the shear-thickening index; ${\dot{\gamma }}_{c}$ is the characteristic shear rate, which corresponds to the shear rate at the viscosity peak; and *d* is the shear-thinning index. Here, *d* > *b* > 0.

III. Model Interpretation

1. At low shear rates ($\dot{\gamma }$ << $\dot{\gamma }$_c_), the model exhibits the following limiting behavior:

(5) $$\eta \approx \eta_{\infty }+\left(\eta_{0}-\eta_{\infty }\right)*e^{A}*{\dot{\gamma }}^{b}$$

Since *b* > 0, the viscosity increases with increasing $\dot{\gamma }$, thereby capturing the shear-thickening behavior.

2. At high shear rates ($\dot{\gamma }$ >> $\dot{\gamma }$_c_), the model exhibits the following limiting behavior:

(6) $$\eta \approx \eta_{\infty }+\left(\eta_{0}-\eta_{\infty }\right)*e^{A}*{{\dot{\gamma }}_{c}}^{d}{\dot{\gamma }}^{b-d}$$

For *d* > *b*, the exponent *b* − *d* is negative (i.e., *b* − *d* < 0), and the viscosity consequently decreases monotonically with $\dot{\gamma }$, consistent with shear-thinning behavior.

3. Critical Point: The viscosity peak occurs in the vicinity of the characteristic shear rate $\dot{\gamma }$_c_.

## Figures and Tables

**Table 1:** Fitting parameters of the new equation constructed based on the Carreau model.

Sample	Concentration wt%	*η*_0_, mpa·s	*η*_∞_, mpa·s	*A*	*b*	$\dot{\gamma }$ _c_	*d*	R^2^
AM/AA/AMPS	0.1	4.213	1.03	5.553	0.915	0.095	1.236	0.9374
	0.3	206.57	3.374	3.731	0.633	0.056	1.186	0.9882
	0.5	3690	23	3.813	0.775	0.042	1.518	0.9977
	0.7	8980.4	36.348	3.162	0.674	0.055	1.519	0.9992
HPG	0.1	4.900	1.849	24.703	17.56	0.234	17.478	0.9581
	0.3	29.985	9.222	27.518	1.556	0.335	1.695	0.9537
	0.5	27.304	10.411	3.461	0.367	0.536	0.787	0.9522
	0.7	930.4	23.414	0.3	0.04	0.216	0.748	0.9987
XG	0.1	10.229	5.45	9.22	1.744	0.052	2.171	0.9073
	0.3	2572	14.9	5.91	1.148	0.03	1.849	0.9943
	0.5	10,896	27.4	8.158	1.636	0.024	2.406	0.9959
	0.7	24,980	35.2	10.483	2.151	0.02	2.968	0.9972

**Table 2:** Hysteresis Loop Area, Pa·s^−1^.

Concentration, wt%	AM/AA/AMPS	XG
0.1	/	0.385
0.3	/	7.07
0.5	1.18	23.73
0.7	2.87	30.36

**Table 3:** Relaxation Time, s.

Concentration, wt%	AM/AA/AMPS	HPG	XG
0.1	/	/	/
0.3	0.183	/	0.031
0.5	0.042	/	0.017
0.7	0.026	/	0.012

**Table 4:** Residual Viscosity and Residual Rate After Heating from 60 °C to 90 °C Within 60 min at a Shear Rate of 100 s^−1^.

Concentration, (wt%)	AM/AA/AMPS		HPG		XG	
	Viscosity, mp·s	Retention Rate, %	Viscosity, mp·s	Retention Rate, %	Viscosity, mp·s	Retention Rate, %
0.1	5.5	56.35	2.16	60.00	5.11	48.57
0.3	22.7	75.67	8.48	36.87	27.5	64.42
0.5	40.71	76.22	36.79	44.12	63.5	70.33
0.7	70.53	85.25	80.97	51.84	114	78.62

**Table 5:** Viscosity (mPa·s) After Heating to 90 °C Within 30 min Followed by Continuous Shearing for 60 min at a Shear Rate of 100 s^−1^.

Concentration (wt%)	AM/AA/AMPS		HPG		XG	
	Viscosity, mp·s	Retention Rate, %	Viscosity, mp·s	Retention Rate, %	Viscosity, mp·s	Retention Rate, %
0.1	3.6	53.73	5.5	50.11	4.7	45.63
0.3	18.8	57.54	9.06	36.92	23.8	53.49
0.5	36.4	75.00	28.51	31.07	41.7	49.30
0.7	60.9	74.26	55.2	32.09	98.6	65.13

**Table 6:** Four-parameter Viscosity–Temperature Relationship Model Parameters of Viscosity–Temperature Curves for Three Systems.

Sample	Concentration, wt%	*η*_0_, mpa·s	*η_min_*, mpa·s	*k*	*C*	R^2^
AM/AA/AMPS	0.1	6.625	3.55	0.062	2.936	0.9869
	0.3	30.97	18.45	0.056	3.343	0.9855
	0.5	47.17	36.13	0.084	3.108	0.9826
	0.7	79.17	60.44	0.066	2.995	0.9775
HPG	0.1	24.43	5.48	0.384	1.021	0.9519
	0.3	31.77	9.05	0.121	1.731	0.9871
	0.5	76.56	28.44	0.084	1.944	0.9906
	0.7	161.77	54.41	0.083	1.833	0.9934
XG	0.1	10.21	4.69	0.069	2.322	0.9934
	0.3	41.59	23.29	0.051	2.703	0.9926
	0.5	86.72	40.69	0.08	1.49	0.9992
	0.7	153.56	98.96	0.065	2.04	0.9981

## Data Availability

The original contributions presented in this study are included in the article Further inquiries can be directed to the corresponding authors.
